# Challenges for Kinetics Predictions via Neural Network Potentials: A Wilkinson’s Catalyst Case

**DOI:** 10.3390/molecules28114477

**Published:** 2023-05-31

**Authors:** Ruben Staub, Philippe Gantzer, Yu Harabuchi, Satoshi Maeda, Alexandre Varnek

**Affiliations:** 1 Institute for Chemical Reaction Design and Discovery (WPI-ICReDD), Hokkaido University, Kita 21, Nishi 10, Kita-ku, Sapporo 001-0021, Japan; 2 Japan Science and Technology Agency (JST), ERATO Maeda Artificial Intelligence in Chemical Reaction Design and Discovery Project, Kita 10, Nishi 8, Kita-ku, Sapporo 060-0810, Japan; 3 Department of Chemistry, Faculty of Science, Hokkaido University, Kita 10, Nishi 8, Kita-ku, Sapporo 060-0810, Japan; 4 Research and Services Division of Materials Data and Integrated System (MaDIS), National Institute for Materials Science (NIMS), 1-1 Namiki, Tsukuba 305-0044, Japan; 5 Laboratory of Chemoinformatics, UMR 7140, CNRS, University of Strasbourg, 67081 Strasbourg, France

**Keywords:** Neural Network Potential (NNP), Artificial Force Induced Reaction (AFIR), Generative Topographic Mapping (GTM), Wilkinson’s catalyst

## Abstract

Ab initio kinetic studies are important to understand and design novel chemical reactions. While the Artificial Force Induced Reaction (AFIR) method provides a convenient and efficient framework for kinetic studies, accurate explorations of reaction path networks incur high computational costs. In this article, we are investigating the applicability of Neural Network Potentials (NNP) to accelerate such studies. For this purpose, we are reporting a novel theoretical study of ethylene hydrogenation with a transition metal complex inspired by Wilkinson’s catalyst, using the AFIR method. The resulting reaction path network was analyzed by the Generative Topographic Mapping method. The network’s geometries were then used to train a state-of-the-art NNP model, to replace expensive ab initio calculations with fast NNP predictions during the search. This procedure was applied to run the first NNP-powered reaction path network exploration using the AFIR method. We discovered that such explorations are particularly challenging for general purpose NNP models, and we identified the underlying limitations. In addition, we are proposing to overcome these challenges by complementing NNP models with fast semiempirical predictions. The proposed solution offers a generally applicable framework, laying the foundations to further accelerate ab initio kinetic studies with Machine Learning Force Fields, and ultimately explore larger systems that are currently inaccessible.

## 1. Introduction

**Figure 1:**
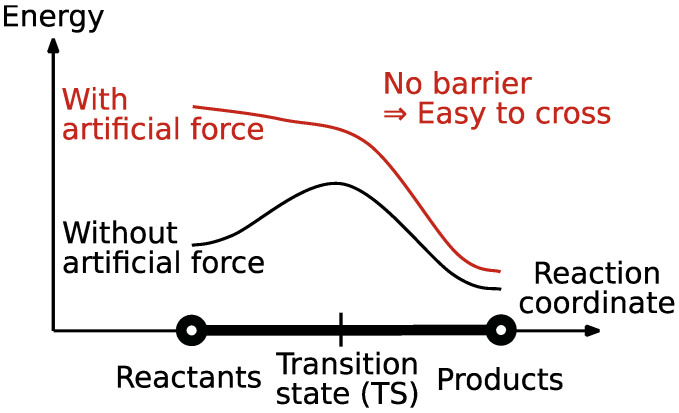
Description of the AFIR method. An artificial force is applied to easily cross reaction barriers.

**Figure 2:**
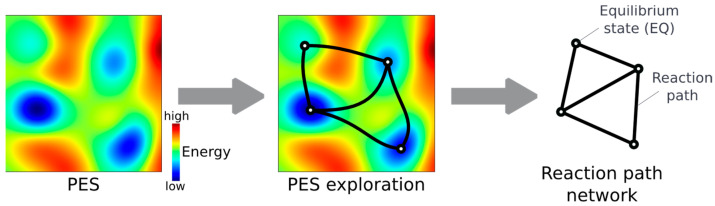
Construction of the reaction path network from the potential energy surface (PES).

Ab initio kinetic studies offer valuable insights into reaction mechanisms [1] through reaction path calculations for elementary steps, providing molecular geometry changes and reaction barrier heights along the reaction path. Reaction paths are defined as the lowest energy path between local minima on the Potential Energy Surface (PES). A convenient and well-established way to search for a path connecting two local minima is the Artificial Force Induced Reaction (AFIR) method [2] (see Figure 1), where an external force is applied to overcome the targeted barrier, producing an approximate reaction path that is then refined. Recently, the development of a systematic reaction path search procedure, based on the AFIR method, has made it possible to systematically search the whole PES for both the minimum energy structures and the reaction paths connecting them [3]. Such a reaction path search produces a complex graph, called a reaction path network, in which the nodes correspond to the stable molecular geometries—equilibrium states (EQs)—and the edges correspond to reaction paths (see Figure 2).

Despite the availability of various algorithms, the visualization of big networks tends to become problematic, due to the large amount of data to represent [4]. Instead of classically representing the reaction network [5], the PES could be visualized on 2-dimensional maps, resulting from encoding molecular structures by a vector, followed by the application of dimension reduction techniques, such as principal component analysis (PCA) [6,7], locally linear embedding [8], multidimensional scaling [9,10,11], and isometric feature mapping [8,9]. In this paper, we propose to use, for the first time, the Generative Topographic Mapping (GTM) [12] approach to represent reaction path networks.

Once the reaction path network is constructed, the species concentration at a given temperature and reaction time could be estimated by solving the system of linear differential equations for reaction rates of elementary reaction steps. The soft clustering method, called Rate Constant Matrix Contraction (RCMC) [13], is used to solve the kinetic simulation, because the numerical integration of sequential differential equations quickly becomes unstable [14]. Combining the RCMC and AFIR methods enables on-the-fly kinetic simulation [15] during the reaction path search, which is used in the kinetic-based navigation method for efficient reaction path exploration of the chemically accessible region.

The main bottleneck of a typical reaction path search comes from the high computational costs of PES assessments using Density Functional Theory (DFT). Even with the kinetic-based method, more than 95% of a search cost arises from gradient calculations. For this reason, a DFT-based search considering all degrees of freedom is usually limited to about 30 atoms. To overcome this limitation, it is possible to use a faster semiempirical potential [16,17], such as xTB [18], for gradient calculations. However, such a semiempirical method is prone to low energy accuracy, especially for transition metal complexes [19]. Yet, inaccurate energy barriers can cause poor reproduction of the reaction kinetics, which often leads to poor kinetic navigation and negatively affects the search efficiency.

In parallel, recent advances in Neural Network Potentials (NNPs) [20,21] offer many examples [22,23,24,25,26,27,28,29] of highly accurate predictions at a significantly lower cost than their corresponding ab initio calculations, provided sufficient training data is available [30,31].

**Figure 3:**
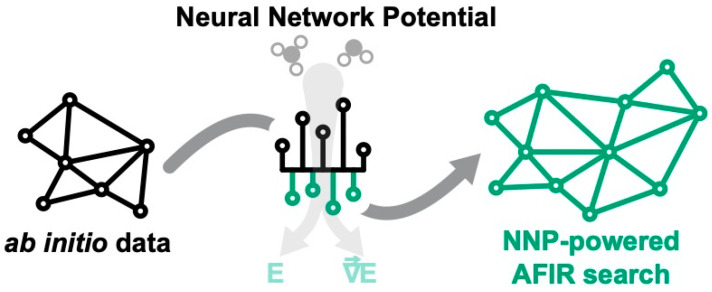
Overview of the novel approach described in this article: using NNP-based models trained on prior ab initio data to support AFIR-based reaction path searches.

Therefore, in this article, we investigate the applicability of NNPs to reaction path search with the kinetic-based navigation method. For this application, we propose to replace expensive ab initio calculations with NNP-based predictions during the search (see Figure 3). This solution combines the search efficiency of the kinetic-based navigation method and the computational efficiency of machine learning-based predictions, but requires an adequately trained NNP model.

It should be noted that, in addition to specialized Neural Network-based models directly predicting reaction kinetics [32,33], NNPs have been used in numerous kinetic studies for fitting the PES [34,35] and accelerating molecular simulations. In such studies, NNPs are typically powering Molecular Dynamics (MD) simulations [36], Well-Tempered [37] metadynamics [38,39], or Nudged Elastic Band-based refinement of reaction path guesses [40], where only small-to-moderate artificial forces are applied. In one recent study, potentially strong artificial forces were applied for the training set construction, but the NNP-powered reaction path search itself was done by typical MD simulations [41]. In contrast, we focus here on the challenges for designing NNP-based models to support AFIR-based reaction path searches, where strong exploration forces are involved.

**Figure 4:**
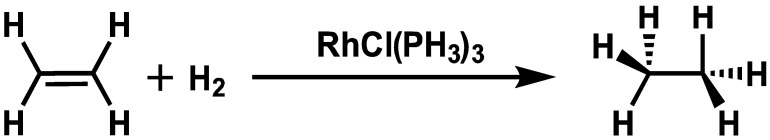
Reaction scheme of hydrogenation using a simplified catalyst, inspired by Wilkinson’s catalyst (i.e., RhCl(PPh_3_)_3_).

For this study, we consider the hydrogenation of ethylene catalyzed by a transition metal complex, inspired by Wilkinson’s catalyst (see Figure 4) [42], because of the authors’ familiarity with this system. First, a preliminary reaction path search is performed at the DFT level, using the kinetic navigation method. The resulting reaction path network is then analyzed with GTM, allowing to describe the different reaction steps and visualize the exploration of the PES during the reaction path search.

We then focus on an NNP-powered reaction path search with the AFIR method and kinetic-based navigation.

After identifying the fundamental robustness issue of general-purpose NNPs in the presence of strong external forces, we focus on designing robust NNP-based models that are capable of generalization across the reaction path network.

We finally investigate the ability of such models to support an NNP-powered search capable of reproducing DFT-based chemical reaction yields, despite being trained on a fraction of the DFT-based reaction path network.

## 2. Materials and Methods

### 2.1. Reaction Path Search Using the AFIR Method

In this study, the single component (SC)-Artificial Force Induced Reaction (AFIR) method [3] is used to search for reaction pathways. AFIR defines two or more groups of atoms, called fragments, in an equilibrium state (EQ), and tracks reaction pathways between different EQs by applying external forces. In the SC algorithm, two atoms are chosen, and the fragments are defined around these atoms; in the SC-AFIR method, transitions between EQs are caused by applying a force that pushes or pulls the two defined fragments around the two atoms. In many reaction systems, this method has so far proven to be a useful tool for searches [43,44]. In complement to the current study, Neural Network approaches were recently proposed [45] to optimize the order in which the forces are applied in SC-AFIR (i.e., the order in which the atomic pairs and the direction of the forces are chosen and calculated), which is a very important factor determining the efficiency of the search.

A kinetic simulation on the constructed reaction path network requires the rate constants of each elementary process. In the present study, the rate constants are defined based on the ΔΔG along the approximate reaction path, relaxed by the locally updated planes (LUP) method [46] (denoted by LUP path). A previous study suggested that the reaction path network of LUP paths (LUP-path network) reproduces adequately the kinetics obtained using the actual transition states [47]. Once all the rate constants corresponding to all edges of the reaction path network are computed, the first-order simultaneous differential equations governing the kinetics can be solved numerically to provide the reaction yields under user-defined conditions. In practice, this kinetic simulation involves stiff equations, with a mixture of very fast and very slow processes, which are difficult to solve efficiently by numerical integration. In contrast, the rate constant matrix contraction (RCMC) method [13] proposed by Sumiya and Maeda is effective, because it allows fast kinetic simulations to be performed by a clustering operation called contraction.

### 2.2. Dataset Description

In this study, instead of the classical Wilkinson’s catalyst RhCl(PPh_3_)_3_, the simplified catalyst RhCl(PH_3_)_3_ has been considered.

The present kinetic study by AFIR search has been performed at the RωB97X-D/Def2-SVP level of theory [48,49]. The details of the reaction path search are written in the supporting information (see Supplementary Materials Section S1). This search produced a reaction path network where each edge is representing a single elementary process explored with the AFIR method. For each elementary process explored, the corresponding LUP path obtained is represented by a set of geometries along the path. For each of these geometries, the potential energy, gradients, and electric dipole moments were computed at the RωB97X-D/Def2-SVP level of theory in this context (see Supplementary Materials Section S1). The results obtained have been compiled into a database, WilkinsonAFIRdb, using the ASE database framework [50].

Alternatively to the present DFT-based AFIR search, one should note that a database of AFIR-generated reaction path networks was recently constructed using quantum chemistry-aided retrosynthetic analysis (QCaRA) [51], which traces back the reaction paths from the target product to various reactant candidates and their theoretical yields [52]. Such a database should also be appropriate to train NNP-based models made to support AFIR-based reaction path searches.

### 2.3. GTM Visualization

Generative Topographic Mapping (GTM) is a dimension reduction method, which allows the visualization of a data distribution on a 2-dimensional map. A more detailed description of GTM underlying algorithms can be found in a previous paper [12]. The main idea of GTM consists in inserting a flexible hypersurface, called manifold, into the high-dimensional descriptor space, with a subsequent projection of these data points into a 2D latent space grid. A data property can be added as a 3rd axis of the 2D map, forming a so-called property landscape [53]. Each landscape position is colored according to the property value; this value is the average property of the data subset projected to that position on the landscape. Here, two types of landscapes are considered: (i) the class landscape, which assigns a color scale to the map depending on the population of one class of compounds compared to that of another class; (ii) the energy landscape, which colors the maps according to potential energy values computed with DFT.

Here, GTM was trained on 10,000 3D geometries randomly selected from the WilkinsonAFIRdb dataset and encoded by 3D pairwise-sorted distance-based descriptors (see Section 2.4). GTM parameters were optimized using the Genetic Algorithm [54]. A manifold was trained by minimizing a cost function, which combined an error of energy prediction and the inverse informational entropy.

### 2.4. 3D Pairwise-Sorted Distance-Based Descriptors

The 3D structures were encoded by descriptors derived from interatomic distances, to avoid the need for alignment [10,30]. These distances were then grouped according to their corresponding atom types and sorted within each group, leading to 3D pairwise-sorted distance-based descriptors. Those descriptors are also invariant by atomic permutations [30]. Such descriptors, or their inverse, were already applied to: identify peptides’ aggregation pathways [55]; analyze proton transfer reactions mechanisms [56]; and predict atomic, potential, and interaction energies [57,58,59,60].

In our implementation, all interatomic distances were first computed for each unique structure and labeled by the atomic numbers of the constituting atoms. The same-labeled distances were then gathered and sorted in ascending order. Finally, those sorted distances were concatenated and formed the descriptor vector for each 3D structure.

The 3D structures corresponding to the same 2D structure were grouped into subsets. Descriptors values characterizing each subset were computed as the Boltzmann-weighted sum of descriptors of related 3D structures, according to Equations (1) and (2):

(1) $$X_{p}=\sum w_{i}X_{ip}$$

(2) $$w_{i}=e^{\left(E_{i}/\left(k_{B}T\right)\right)}/(\sum \;e^{\left(E_{i}/\left(k_{B}T\right)\right)},$$

where *X_p_* and $X_{ip}$ are the *p*-th term of the descriptor characterizing, respectively, the entire subset and the *i*-th structure, *i* iterates over 3D structures belonging to the same 2D structure, $w_{i}$ is the weight associated to the structure *i*, $E_{i}$ is the relative potential energy of *i* compared to the lowest energy observed within the reaction path network, $k_{B}$ is the Boltzmann constant, and T=300 K is the considered temperature.

### 2.5. Neural Network Potential Architecture

For this study, we are considering a Neural Network Potential, as recommended for efficiently handling large amounts of training data [61,62]. We have chosen the publicly available SpookyNet [22] architecture for its enhanced description of non-local effects via a dedicated attention network [63], and its inclusion of physics-inspired additional terms. SpookyNet is a general-purpose NNP based on graph convolutional networks, where the predictions are composed of an attention-based non-local part, and a local part based on atomic descriptions, which are iteratively refined via interaction modules acting as convolutional filters with neighboring atomic environments.

### 2.6. NNP(+xTB) Models

In addition to a Graph Convolutional Neural Network architecture, SpookyNet models also include additional terms by default. So, the predicted energy is composed of 4 terms (gradients and Hessian predictions are analytically derived from the energy):

(3) $$E_{SpookyNet}=E_{NN}+E_{ZBL}+E_{D4}+E_{elec},$$

where $E_{NN}$ is the Neural Network based atomic energy predictions, $E_{ZBL}$ is a repulsion energy term from a Ziegler-Biersack-Littmark (ZBL) potential [64] with learnable parameters, $E_{D4}$ is the D4 dispersion correction [65], and $E_{elec}$ is an electrostatic term using partial charges predicted along with the atomic energies. Note that all terms have learnable parameters.

**Figure 5:**
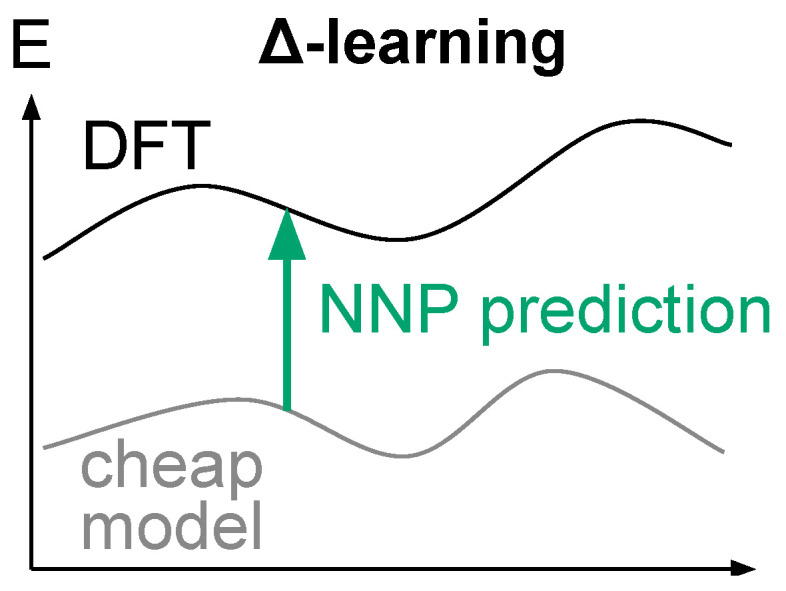
Δ-learning scheme for improving model predictions.

Such an approach can be seen as an instance of Δ-learning [66], where model predictions are complemented by an external potential, so that the model can focus on learning only the difference (hence the Δ-learning name) between the target property and the external potential, see Figure 5.

For NNP(+xTB) models, we replaced the default additional terms with predictions from the GFN2-xTB [67] semiempirical potential (simply referred to as xTB):

(4) $$E_{NNP\left(+xTB\right)}=E_{NN}+E_{xTB},$$

The additional terms of SpookyNet (i.e., $E_{ZBL}$, $E_{D4}$ and $E_{elec}$) were not kept for NNP(+xTB) models, because the GFN2-xTB potential already includes a repulsion energy term and a dispersion energy term, based on the D4 dispersion model.

## 3. Results

### 3.1. Reaction Path Network for Hydrogenation Using a Simplified Wilkinson’s Catalyst

**Figure 6:**
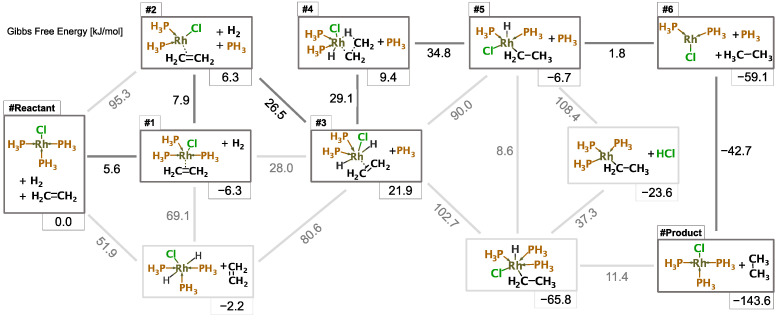
Reaction path network obtained with the AFIR method and kinetic-based navigation, computed at the DFT level. Boxes represent equilibrium structures; edges represent reaction paths. The main reaction steps (depicted in dark grey) correspond to intermediate structures #1–6.

Figure 6 shows the kinetically important 2D structures of the reaction path network obtained in this study at the DFT level. In this figure, only the lowest reaction barriers between groups are shown, assuming that the reaction barriers within groups are sufficiently low. In this reaction, the leftmost group represents the reactants. The group with the highest yield, at 300 K, represented in the bottom right-hand corner, is the one containing ethane. The WilkinsonAFIRdb database contains the electronic energy, gradients, and electric dipole moments for 118,240 geometries, including 6298 approximate transition states (TS) and 2049 equilibrium states (EQ). These geometries correspond to the reaction paths for the 6298 elementary processes explored with the AFIR method.

The traditional hydrogenation of alkenes by H_2_, catalyzed by the Wilkinson’s catalyst (i.e., RhCl(PPh_3_)_3_), involves the following steps after the PPh_3_ dissociation: oxidative addition of H_2_ to the metal complex; alkene coordination; alkene insertion; and reductive elimination of alkane [68].

Koga et al. [69] computationally studied the hydrogenation of ethylene with a simplified catalyst RhCl(PH_3_)_2_, and reported that the oxidative addition of H_2_ occurs before the ethylene coordination. In the present study, we have considered the non-dissociated simplified catalyst RhCl(PH_3_)_3_ by explicitly modeling all three PH_3_ ligands. For this system, we found that the ethylene coordination is the first step of the hydrogenation, producing RhCl(PH_3_)_3_(C_2_H_4_); followed by the dissociation of a PH_3_ ligand; then, the oxidative addition of H_2_, ethylene insertion, and reductive elimination of ethane proceed with two PH_3_ ligands. Finally, the initial RhCl(PH_3_)_3_ catalyst is restored, completing the catalytic cycle.

Experimentally, ethylene is a well-known poison of Wilkinson’s catalyst, leading to the formation of RhCl(PPh_3_)_2_(C_2_H_4_), which is not active enough to react with H_2_ at 1 atmosphere, likely due to the large π-acidity of the ethylene ligand [42]. This observation seems consistent with the preferential initial coordination of ethylene in the present study, even though further research is required to understand if the mismatch on the number of coordinated phosphines (2 × PPh_3_ vs. 3 × PH_3_) is solely due to steric effects. Unlike Wilkinson’s catalyst, we found that the hydrogenation of ethylene can proceed with the simplified catalyst. This outcome could be partially due to a lower (compared to the original PPh_3_) simulated π-acidity of the PH_3_ ligands [70], especially since this property was found to be particularly sensitive to the accuracy of the P 3d orbitals description [71] (see Supplementary Materials Section S8 for an in-depth analysis). However, such analysis is out of the scope of the present study. In general, for practical machine learning applications, the quality of the dataset is essential. To this end, it is desirable to find the most adapted level of theory by performing comparison against higher precision calculations, such as CCSD(T)-F12 [72,73]. One should note that our method is a priori, compatible with any level of theory.

### 3.2. Data Visualization with GTM

**Figure 7:**
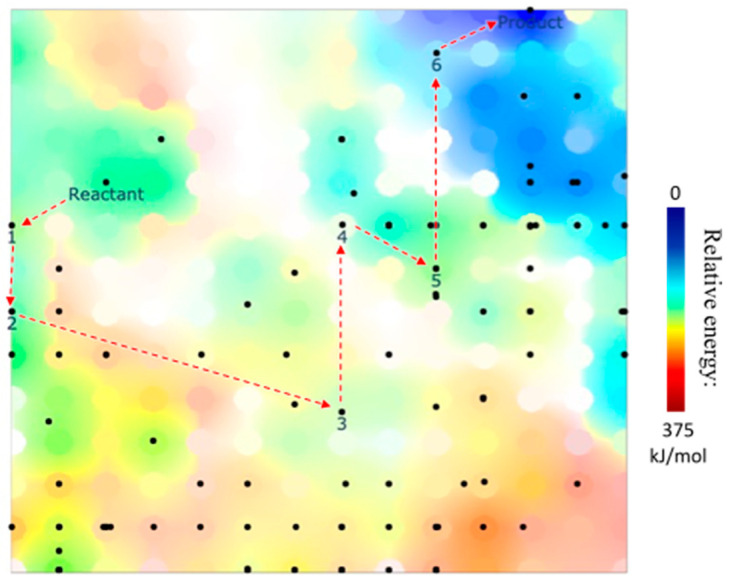
GTM energy landscape for the entire DFT-based network, with projected groups. Groups belonging to the proposed hydrogenation reaction path are labeled, where numbers correspond to the reaction intermediates involved, in order. The corresponding reaction path is highlighted by red arrows.

**Figure 8:**
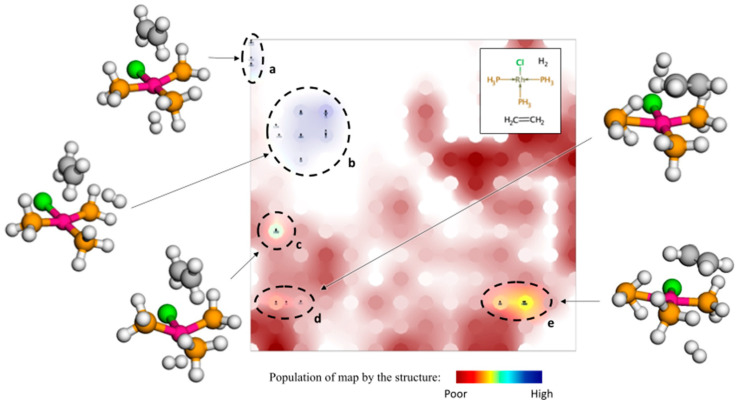
GTM class landscape showing distribution of 3D structures corresponding to reactants. 5 different clusters (labelled with letters) can be identified and correspond to distinct conformations.

The DFT data were visualized on the GTM energy and class landscapes (Figure 7 and Figure 8). On the energy landscape (Figure 7), black dots characterize different 3D EQs groups associated with their related 2D structures (see Section 2.4). The main reaction path 1–6 structures are situated in the low or medium energies areas.

Class landscape (Figure 8*)* displays the distribution of 58 EQs, forming the reactant group, populating 5 areas of the map. In structures situated in low and middle-energy areas *a*–*c* with, respectively, 6 and 26 structures, the rhodium atom coordinates all 3 phosphorus atoms of PH_3_ groups; whereas, in high-energy areas *d* and *e*, one of the PH_3_ groups is oriented toward the rhodium by its hydrogen atoms. The latter areas correspond to some sort of dead-end of the reaction network. Class landscapes demonstrating distribution of 3D structures of the product and those formed in main reaction steps 1–6 are given in Supplementary Materials Figure S11. Notice that, thanks to the Boltzmann-like weighting of descriptors, the representative projection of each group is always located near its lowest-energy cluster.

**Figure 9:**
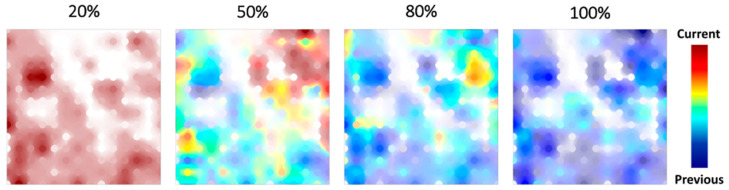
GTM landscapes describing first 20%, 50%, 80%, and 100% of the network exploration discovered in the DFT-based search. Each next map visualizes a class landscape, where the brown color corresponds to the zones populated exclusively by “new” (with respect to the previous map) structures, and the blue color—to the zones populated by “old” structures. Notice that the map accommodating the first 20% contains only “new” structures.

In order to analyze the reaction network expansion, we have compared GTMs with projected first 20%, 50%, 80%, and 100% structures discovered in the DFT-powered search (Figure 9). One can see that the first 50% of the network already covers the apparent chemical space of the entire network. Indeed, the map accommodating 80% of data does not contain purely brown zones; the new (compared to map of 50%) structures populate mostly the products zone.

### 3.3. Applicability of Neural Network Potentials to AFIR-Based Reaction Path Search

In this study, we make use of the data generated at the DFT level during the AFIR-based reaction path search, to study the applicability of NNP-based models to replace DFT predictions during an AFIR-based reaction path search (i.e., supporting an NNP-powered AFIR-based reaction path search).

#### 3.3.1. NNP Performance on Pre-Obtained Geometries

**Figure 10:**
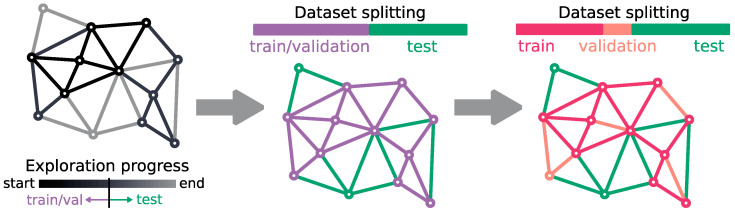
Dataset splitting scheme into training set, validation set, and test set. First, a search-related timestamp is chosen (e.g., when 50% of the network’s paths has been explored by the search, which is equivalent to: when the search is half-completed). The geometries corresponding to paths already explored before this timestamp are grouped into the train/validation set, and the test set is composed of all geometries corresponding to paths that were not yet discovered at this time of the search. The train/validation set is then split into a training set and a validation set randomly, while ensuring that all geometries corresponding to a single path are either within the training set or the validation set (i.e., validation geometries correspond to paths which are not covered in the training set, except for the EQs shared with training paths).

We have trained SpookyNet models on the geometries along the IRC paths of the reaction path network explored during the DFT-based reaction path search, described in Section 3.1. For this study, we have designed a future-oriented testing, by training the model on the earlier paths explored during the DFT-based search and evaluating the model predictions on the remaining paths (see Figure 10 and Supplementary Materials Section S4 for more details on the train/validation/test splitting).

**Figure 11:**
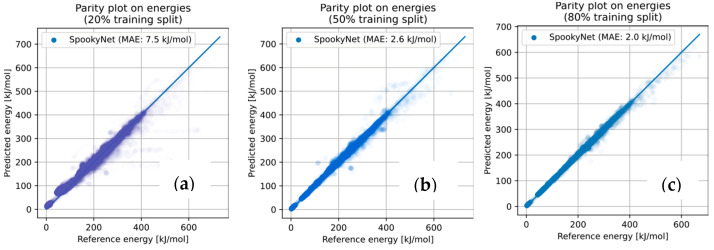
Performance of SpookyNet models trained on the first paths explored during the DFT-powered AFIR-based reaction path search. The energy predictions and energy references (i.e., DFT energies) are displayed for the remaining geometries, with transparency for better readability. (**a**) Model trained on the first 20% of paths explored, the remaining 80% are represented, *R*^2^ = 0.97; (**b**) Model trained on the first 50% of paths explored, the remaining 50% are represented, *R*^2^ = 0.995; (**c**) Model trained on the first 80% of paths explored, the remaining 20% are represented, *R*^2^ = 0.998.

We have considered different training sizes (using only the first 20%/50%/80% of the paths explored during the search), as well as multiple training techniques (ensemble [74], dropouts [75], Δ-learning [66], …) as described in Supplementary Materials Sections S3 and S5. All the resulting models consistently showed predictive capabilities to accurately reproduce energies on the later parts of the DFT-based search, despite being trained only on the earliest paths discovered, see Figure 11. Thus, a SpookyNet model, trained only on the first 20% of paths explored, achieved a Mean Absolute Error (MAE) of <8 kJ/mol on the energy predictions for the remaining geometries. Using the first 50% or more of paths for training led to models achieving chemical accuracy (MAE < 3 kJ/mol on the geometries of the remaining paths).

#### 3.3.2. Reaction Path Search Using the NNP Model

In light of these promising preliminary results, we have developed an efficient interface between the GRRM program and an NNP-based model, which enables NNP-powered AFIR-based reaction path searches, where all energies and gradients evaluations are performed with the trained NNP.

For the first NNP-powered AFIR-based search, we have considered a local exploration around the most stable conformer of the reactants. This area is expected to have been particularly well-explored during the DFT-based search. Therefore, one could expect that the resulting training data is well-adapted to this local search (even when using only the first 20% of paths explored during the DFT-based search, since the starting point of the DFT-based search is located in this region).

**Figure 12:**
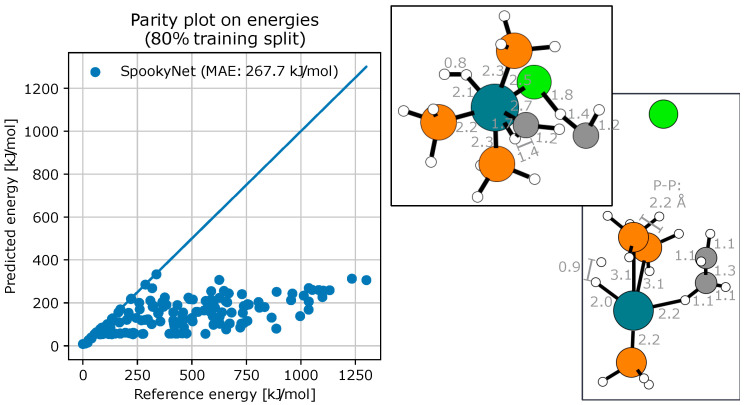
Performance of SpookyNet when powering a local AFIR-based exploration around the most stable reactants conformer. Each point represents a PES stationary point geometry (i.e., an approximate TS or EQ) obtained during the search. The energy predictions are generated during the search, and the energy references (i.e., DFT energies) are computed a posteriori. Here, the largest errors were found on structures with no apparent steric clashes, but with dissociated structures and/or isolated atoms. The model was trained on the first 80% of paths explored during the preliminary DFT-powered search, *R*^2^ = −76.

Despite these considerations, all trained SpookyNet models performed surprisingly badly, regardless of the training set size, see Figure 12. Such a poor performance (contrasting with the performance of these same models on pre-obtained geometries) indicates a strong discrepancy between the geometries generated by the DFT-based search and those from the NNP-based local searches.

In particular, we observed a serious energy underestimation of broken geometries (dissociated structures, steric clashes, broken valences, …). These results illustrate a dramatic lack of robustness of the trained models for supporting AFIR-based explorations. Indeed, by incorrectly evaluating broken geometries as stable, the fitted potentials contribute to drag the AFIR-based search toward unphysical pathways.

We attribute this failure to the combination of three distinct factors:

- *Lack of physics*: While general-purpose NNPs, such as SpookyNet, do respect fundamental symmetries (translation, rotation, …), their functional forms (i.e., the mathematical models) are not physics-based. In particular, their asymptotic behavior is not governed by physical principles. Although SpookyNet models already include additional trainable terms that are physics-inspired (*E_ZBL_*, *E_D_*_4_ and *E_elec_*), these terms do not seem sufficient to ensure physical asymptotic behavior outside the training domain.
- *Training bias*: Due to the aforementioned lack of physics, the NNP considerably relies on the training data, yet the dataset does not contain strongly broken geometries. Indeed, such geometries are not encountered during the DFT-based search, because all paths leading to them would be rightfully assessed as too high in energy for the exploration to continue. Therefore, the trained NNPs cannot properly handle these extreme geometries, leading them to be poorly described.
- *Strong exploration forces*: Even if sufficient training data is available in the accessible valleys of a potential energy surface (i.e., chemically reasonable geometries), we believe that applying a strong external force can drive a properly described system outside the locally well-defined valleys of the fitted potential.

#### 3.3.3. Δ-Learning Solution for Robust NNP-Based Models

Let us quickly examine what can, or cannot, be done about the three factors aforementioned:

- Strong exploration forces are a powerful tool to efficiently sample rare events [76], so we believe that one should focus on designing models that can support them, instead of removing them.
- SpookyNet models need to be trained on broken geometries to properly describe them. We argue that complementing the training dataset a priori with broken geometries is not reasonable, because one cannot easily predict in advance the pitfalls of a fitted potential, and one cannot reasonably include all possible broken geometries in the training set. A simple argument to convince the reader is to consider N atoms randomly distributed in a box: the probability that the resulting geometry is chemically reasonable is close to zero, therefore illustrating the inconceivably large ratio of broken geometries over reasonable geometries. We further argue that such training bias toward reasonable geometries in available datasets is actually desirable, because we believe it is unreasonable to waste computational resources on unreasonable geometries.

This leaves only the lack of physics to consider, which we are proposing to tackle via Δ-learning. Δ-learning is a well-known technique to improve the accuracy of a model [77,78,79]. Uncommonly, we are here considering this technique to enhance the robustness of our model.

In the context of this article, we are formulating two main hypotheses concerning the inclusion of physics-based principles via Δ-learning:

**Hypothesis 1 (H1).**  *Sufficiently robust models can be achieved by complementing SpookyNet-based NNPs with physics-based additional terms*.

**Hypothesis 2 (H2).**  *In that regard, a robust standalone external model is better than the default trainable additional terms (E_ZBL_, E_D4_ and E_elec_)*.

The fundamental benefit of the proposed Δ-learning solution is to be virtually applicable to any general-purpose NNP architecture. Therefore, we avoid the inconvenience of designing a novel NNP architecture just for AFIR-based search applications. Instead, we propose a general model-agnostic future-proof solution which should, hopefully, also be applicable to the alternatives and successors of SpookyNet.

**Figure 13:**
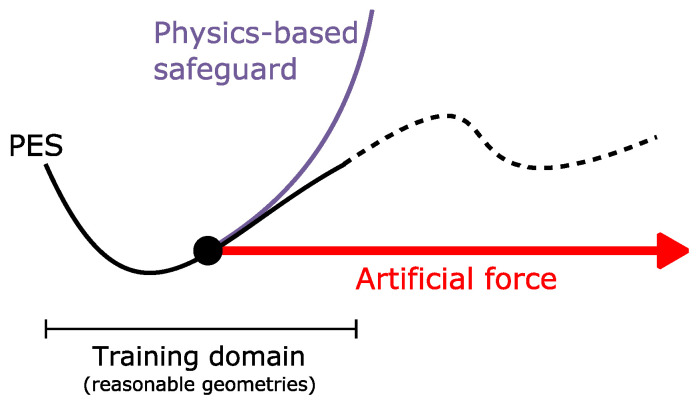
Principle behind the Δ-learning solution as a physics-based safeguard. The physics-based term included via Δ-learning serves as a safeguard to prevent reaching broken geometries due to the AFIR force.

Actually, SpookyNet models are already infused with the concept of Δ-learning, via the introduction of trainable additional terms. In accordance with Hypothesis 2, we propose to replace these additional terms with predictions from an external semiempirical model. We found that the GFN2-xTB potential represents an acceptable compromise between robustness and speed, via its ability to recognize broken geometries while requiring a fraction of the cost of a typical DFT calculation [67]. For the resulting NNP(+xTB) models, the energy prediction follows Equation (4). The idea behind the proposed NNP(+xTB) model is to use a semiempirical method as a continuously derivable safeguard, allowing not only to improve accuracy, but also robustness, in accordance with Hypothesis 1. Indeed, we rely on the xTB part being able to identify broken geometries, even if those geometries are not included in the training set, therefore restraining the AFIR-based search to non-broken geometries (which should be covered by the training set, if the latter was sampled adequately). See Figure 13 for an illustration of this idea.

**Figure 14:**
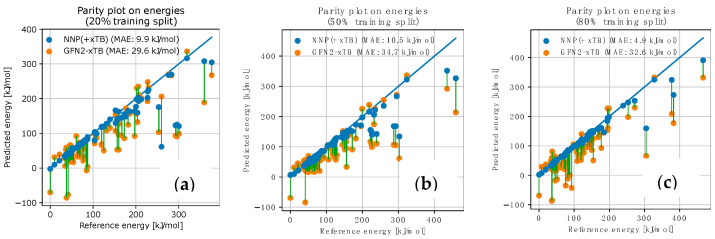
Performance of NNP(+xTB) models when powering a local AFIR-based exploration around the most stable reactants conformer. Each point represents a PES stationary point geometry (i.e., an approximate TS or EQ) obtained during the search. The energy predictions are generated during the search, and the energy references (i.e., DFT energies) are computed a posteriori. xTB and NNP(+xTB) predictions for the same geometry are connected by a line: a red line, if xTB only is closer to DFT, and a green line, if the NNP contribution is beneficial. The energies potentials are shifted to match each other on the WilkinsonAFIRdb dataset. (**a**) Model was trained on the first 20% of paths explored during the preliminary DFT-powered search, *R*^2^ = 0.74; (**b**) Model trained on the first 50% of paths explored, *R*^2^ = 0.79; (**c**) Model trained on the first 80% of paths explored, *R*^2^ = 0.93.

We trained NNP(+xTB) models as previously, and we performed a similar local AFIR-based exploration as before, but powered it by an NNP(+xTB) model instead of a pure NNP (i.e., SpookyNet) model. The new results are presented in Figure 14.

**Figure 15:**
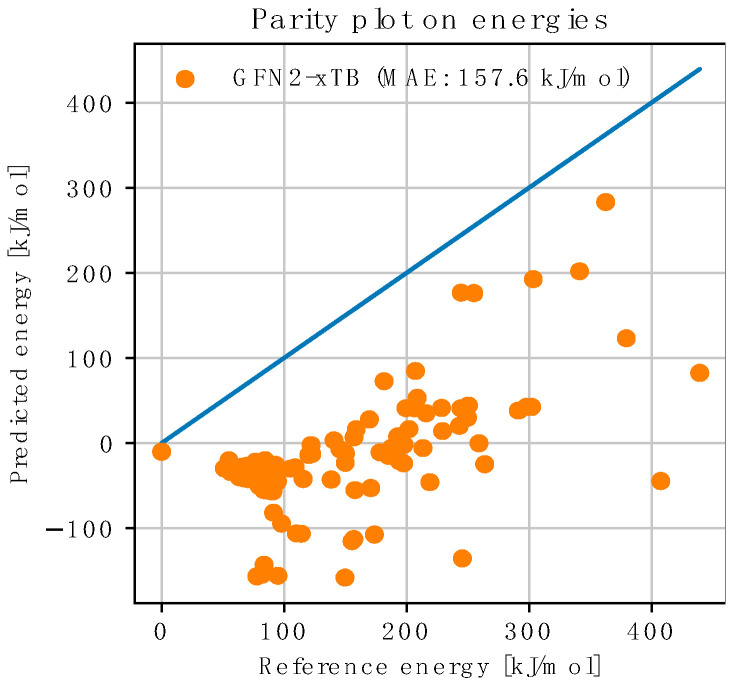
Performance of GFN2-xTB when powering a local AFIR-based exploration around the most stable reactants conformer. Each point represents a PES stationary point geometry (i.e., an approximate TS or EQ) obtained during the search. The xTB energies are generated during the search, and the energy references (i.e., DFT energies) are computed a posteriori. As always, xTB energies are shifted by the exact same amount that was used to minimize the Mean Square Error on the WilkinsonAFIRdb dataset, *R*^2^ = −6.

First of all, we observe that the added xTB term behaves, indeed, as a safeguard, locally preventing the exploration of strongly broken geometries (i.e., very high DFT energy), as illustrated by the difference in the range of the recomputed DFT energies between Figure 12 and Figure 14. In terms of accuracy, we observe that the NNP corrections to the xTB predictions are almost always beneficial, even for most outliers, where the trend is still correct, leading to an MAE around 10 kJ/mol on the predicted energies, despite using the smallest training set size (compared to an MAE > 30 kJ/mol for the uncorrected xTB predictions). Interestingly, a local AFIR-based search powered by xTB only (i.e., no NNP correction) performed significantly worse (see Figure 15), indicating a strong synergy between the two components of the NNP(+xTB) model.

#### 3.3.4. Kinetic Study from Reaction Path Search Using NNP(+xTB)

Strongly shown from these promising local results, we performed a (global) NNP(+xTB)-powered AFIR-based reaction path search (similar to the preliminary DFT-based search), using the same trained NNP(+xTB) models. From these explorations, the resulting reaction yields predicted, and the main products predicted, are reported in Table 1.

We observe a large sensibility of the global yield on the amount of training data used: when using the smallest training set (only the first 20% of paths explored during the DFT-based search), the predicted reaction yields are 0% at all temperatures. This erroneous prediction indicates that severe energy barrier errors were encountered during the NNP(+xTB)-powered AFIR search. In addition, we identified a “leaky holes” behavior [80,81] (i.e., unphysical localized collapses of the potential energy surface) affecting very high-energy pathways, where broken geometries are incorrectly evaluated as very stable (see Figure S7), resulting in the AFIR-based search being dragged toward these unphysical “holes” (due to the optimization nature of the AFIR method). See Supplementary Materials Section S7 for additional details.

In contrast, using NNP(+xTB) models trained on more data, we managed to recover the DFT-based yields at T ≥ 300 K, indicating that neither “leaky holes” capturing the global yields nor severely erroneous energy barriers were found during the search. This result suggests that the discovered reaction path network is well-sampled by the first ≥50% of paths explored during the DFT-based search.

Incidentally, this analysis is in accordance with the GTM observation that the first 50% of the DFT-obtained paths are covering the whole DFT-based reaction path network explored (see Section 3.2). Indeed, if we assume that the final NNP(+xTB)-based network is similar to the converged DFT-based network, then the first 50% of the DFT-obtained paths are also covering the final NNP(+xTB)-based network.

The incomplete reproduction of the yields at 250 K suggests accuracy issues that can be resolved with more training data, as illustrated by the better yields obtained using the largest training set. In any case, the performance of properly trained NNP(+xTB) models was found to be far superior to xTB, only for supporting AFIR-based reaction searches.

## 4. Conclusions

Ab initio kinetic studies typically incur large computational costs, and we found that cheaper semiempirical methods, such as GFN2-xTB, are sometimes not accurate enough to reproduce the reaction kinetics, therefore misleading kinetic-based heuristics. We have proposed to replace expensive ab initio calculations with fast Neural Network Potential predictions during the search. In a case study of hydrogenation of ethylene, catalyzed by a transition metal complex inspired by Wilkinson’s catalyst, we discovered that typical general-purpose NNP models were not robust enough to support an AFIR-based reaction path search, where strong exploration forces were involved. For this reason, NNP predictions were complemented with xTB calculations via Δ-learning. The resulting NNP(+xTB) models could reproduce reaction yields when powering an AFIR-based search, as long as sufficient training was achieved. The Generative Topographic Mapping technique was found to be particularly useful to follow the exploration of the chemical space during the search and identify the zones corresponding to the different steps of the reaction.

We believe that kinetic studies can benefit much from the recent developments in the NNP field. In that regard, the promising performance of our NNP(+xTB) solution is highlighting the importance of robustness for designing adapted potentials.

## Figures and Tables

**Table 1:** Predicted yields at different temperatures, from AFIR-based reaction path search using different potentials.

Predicted Yield	GFN2-xTB	NNP(+xTB) 20% Training	NNP(+xTB) 50% Training	NNP(+xTB) 80% Training	DFT
250 K	0.50%	0.00%	2.09%	31.42%	98.47%
300 K	1.42%	0.00%	96.47%	100%	100%
350 K	2.79%	0.00%	99.95%	99.98%	100%

## Data Availability

The WilkinsonAFIRdb dataset and all data related to the figures can be downloaded at https://doi.org/10.5281/zenodo.7861665 (accessed on 25 May 2023). The source codes (GRRM-NNP interface, training scripts, plotting scripts, …), trained models, GTM maps, and GRRM input files and outputs can be obtained from the authors upon reasonable request.

## Supplementary Materials

The following supporting information can be downloaded at: https://www.mdpi.com/article/10.3390/molecules28114477/s1, Figure S1: Example of GFN2-xTB calculation failure; Figure S2: Dataset training scheme; Figure S3: Influence of cold restarts; Figure S4: Influence of dropout rate; Figure S5: Influence of ensemble learning; Figure S6: Influence of additional terms; Figure S7: Performance of NNP(+xTB) model (20% training) on global AFIR-based search; Figure S8: Example of unphysical geometry generated; Figure S9: Graphical discussion; Figure S10: GTM visualization of early AFIR exploration; Figure S11: GTM class landscapes; Table S1: Relationship between ensemble disagreement and prediction error; Table S2: Main products predicted. Refs [13,22,50,69,71,75,82,83,84,85] are cited in Supplementary Materials.
