# Supplementary material for: Challenges for Kinetics Predictions via Neural Network Potentials: A Wilkinson’s Catalyst Case
Source: Molecules. 2023 May 31;28(11):4477. doi: 10.3390/molecules28114477 (PMC10254369; doi:10.3390/molecules28114477)
Supplement: Supplementary file 1 [file molecules-28-04477-s001.zip › molecules-2352626-supplementary.pdf]

Supporting Information

Challenges for Kinetics Predictions via Neural Network Potentials: a study on Wilkinson's catalyst

Ruben Staub[1,*], Philippe Gantzer[1], Yu Harabuchi,[1,2,3*] Satoshi Maeda[1,2,3,4*] and Alexandre Varnek[1,5*]

[1] Institute for Chemical Reaction Design and Discovery (WPI-ICReDD), Hokkaido University, Kita 21, Nishi 10, Kita-ku, Sapporo, Hokkaido 001-0021, Japan

[2] JST, ERATO Maeda Artificial Intelligence in Chemical Reaction Design and Discovery Project, Kita 10, Nishi 8, Kita-ku, Sapporo, Hokkaido 060-0810, Japan

[3] Department of Chemistry, Faculty of Science, Hokkaido University, Kita 10, Nishi 8, Kita-ku, Sapporo, Hokkaido 060-0810, Japan

[4] Research and Services Division of Materials Data and Integrated System (MaDIS), National Institute for Materials Science (NIMS), Tsukuba, Ibaraki 305-0044, Japan

[5] Laboratory of Chemoinformatics, UMR 7140, CNRS, University of Strasbourg, 67081 Strasbourg, France

* Correspondence: (R.S.) ruben.staub@icredd.hokudai.ac.jp, (Y.H.) y_harabuchi@sci.hokudai.ac.jp, (S.M.) smaeda@eis.hokudai.ac.jp, (A.V.) varnek@unistra.fr

S1. Computational details of the reaction path calculations with the kinetic navigation
S2. Computational details of the local AFIR-based explorations
S3. NNP training details
S4. Details of dataset splitting for NNP training
S5. Influence of NNP training techniques on prediction performances
S6. Predicted main products
S7. Leaky holes behavior
S8. Additional analyses
S9. Detailed conclusions and perspectives
S10. Additional GTM-related analyses

S1. Computational details of the reaction path calculations with the kinetic navigation

A DFT-based SC-AFIR search was applied to the system composed of ethylene (C_2H_4), dihydrogen molecule (H_2) and a simplified Wilkinson catalyst ($ClRh(PH_3)_3$). The RωB97X-D functional and the Def2-SVP basis set were used in the DFT level with Grid=FineGrid option in Gaussian 16 program package. In total, 1863042 gradients calculations and 46595 Hessian calculations were performed during this DFT-based SC-AFIR search. To prevent a molecule from going too far away from the reaction center, a weak force with γ = 100/[N×(N−1)/2] kJ/mol was applied to all atom pairs, where N corresponds to the number of atoms in each system. The reaction path search started from one hundred initial structures composed of the three reactant molecules randomly oriented. The target atoms of the SC-AFIR search were set to all the atoms except the 9 hydrogens of the three PH_3 groups. The model collision energy parameter, γ, was set to 300.0 kJ/mol. The obtained AFIR paths tend to pass through near-TSs of the corresponding reaction pathways (AFIR paths), and further optimization of the AFIR path by the locally updated planes (LUP) method gives a reaction path (denoted by LUP path). During the SC-AFIR searches, the EQ to which the next SC-AFIR procedure was applied was chosen by a kinetic-based navigation method based on the rate constant matrix contraction (RCMC) method. For the kinetics-based navigation, an initial population of 1% was assigned to each generated initial random structure, and conditions of the kinetic simulations were set to 3600.0 s, with temperatures of 250, 300, and 350 K. Kinetic analyses were performed based on the Gibbs energies of the LUP path network. Gibbs energy was estimated by harmonic vibrational analysis, whereby all harmonic frequencies smaller than 50 cm^{-1} were set to 50 cm^{-1}. The search was terminated when a list of EQs with the 130 largest traffic volume values was not updated in the last 130 path calculations. The traffic volume is an index indicating influx/outflux of the population to/from each node during the equilibration [1].

For the construction of the WilkinsonAFIRdb dataset, the RePath mode of the GRRM package was used with the DontLUPOptimization option to compute the energy and gradients of the stored geometries for each relaxed LUP path obtained from the search. In addition, all the energies and gradients computed during the relaxation of the AFIR paths (i.e., the LUP method) can also be kept with the SaveDataAtAllPoints=2 GRRM option and used for dataset construction. However, this alternative approach is expected to give redundant data.

In addition, AFIR-based reaction path searches were performed with NNP(+xTB) models trained on the WilkinsonAFIRdb dataset, using an in-house GRRM-NNP interface. These AFIR-based searches only differ from the preliminary DFT-based AFIR reaction path search (described above) by the potential used for energies/forces predictions, and the stopping criteria. In order to maintain a similar and consistent amount of exploration, we are stopping the search when at least 1708 paths are explored, corresponding to the number of paths explored by the DFT-based AFIR reaction path search.

S2. Computational details of the Local AFIR-based explorations

Local AFIR-based explorations were performed with NNP models (or NNP(+xTB) models) trained on the WilkinsonAFIRdb dataset, using an in-house GRRM-NNP interface. These explorations were performed around the most stable conformer of the reactants found during the DFT-powered AFIR-based exploration. In this local (FirstOnly) mode, GRRM only explores the edges of the complete reaction path network that are adjacent to a specific node (i.e., the most stable conformer of the reactants, in this case).

In this mode, the exploration is systematic (which is otherwise impossible due to the combinatorial nature of the complete AFIR-based reaction path network), allowing for a more meaningful comparison of local explorations using different potentials.

For accuracy evaluation purposes, all equilibrium geometries and transition states obtained during this search were re-evaluated by single point calculation at the RωB97X-D/Def2-SVP level of theory (same as for the WilkinsonAFIRdb dataset). This verification was performed using the ReEnergy mode of the GRRM program.

S3. NNP training details

Training parameters

The WilkinsonAFIRdb database was used to train SpookyNet models with the recommended training parameters from [2], with equal contribution of the energy loss, gradient loss and dipole loss to the total loss function. A notable exception from these recommendations is that the validation set performances were evaluated after each epoch, for more consistency across different training set sizes. The dipole loss was not considered in the loss function if the default E_{elec} additional term of SpookyNet was not used (*e.g.*, NNP(+xTB) models training was done on energy and gradients alone).

The default hyperparameters were used for the SpookyNet models, as they provided sufficient prediction performance in our tests.

Prior to training a model, a reference energy was taken as to cancel the mean energy prediction error of the untrained, and zero-initialized, model over the whole WilkinsonAFIRdb dataset. This reference energy was subtracted from the DFT energies during training and added to the prediction energies during inference. This energy shift provides better convergence for the training and reduces floating-point errors related to the use of single precision arithmetic for the prediction (faster operations on GPU), as well as simplifying the comparison of energies between different potentials (DFT, xTB, NNP).

The trainings were performed on an NVIDIA A100 (40GB) GPU, with a batch size of 100 geometries. Model inferences were performed on CPU.

Data curation

The geometries considered from the DFT-powered search (i.e., in WilkinsonAFIRdb) come from relaxed LUP paths, at DFT level, without the AFIR force. Therefore, one can expect high quality data, since outliers are already managed by the GRRM package during the DFT-powered search.

Crude data curation was performed by whether GFN2-xTB calculations converged: only one GFN2-xTB calculation failed, and the corresponding geometry was excluded from the NNP(+xTB) models trainings (see Figure S1).

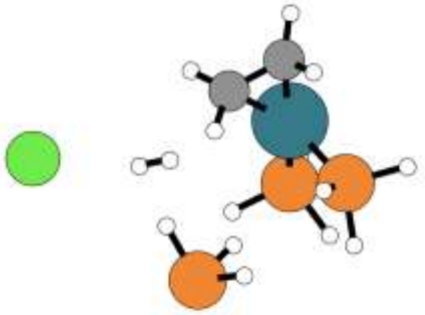

Figure S1. The single geometry for which GFN2-xTB failed. This dissociated structure (high energy at DFT level) was excluded from the WilkinsonAFIRdb.

Additional data curation was performed automatically via GFN2-xTB gradients predictions, excluding geometries whose error on the xTB gradients is larger than 100 times the gradients themselves:

$$\sum_i^N\left(\left\|g_i^{xTB}-g_i^{DFT}\right\|\right) > 100 \times \sum_i^N\left(\left\|g_i^{DFT}\right\|\right), \qquad \text{(S1)}$$

This automatic data curation did not find any geometry outliers.

In addition, an automatic outlier filter was applied during training: a geometry was excluded from the training batch if the root mean squared loss on its gradients was higher than 10 eV:

$$\frac{1}{N}\sum_i^N\left(\left\|g_i^{pred}-g_i^{DFT}\right\|\right) > 10\,\text{eV}, \qquad \text{(S2)}$$

S4. Details of dataset splitting for NNP training

The database was split into a training set, validation set and test set, such that the geometries in the test set correspond to reactive events that have been explored by the AFIR search after the ones from the training/validation sets. In other words, the training/validation sets correspond to the earlier steps of the search, while the test set correspond to the later steps of the AFIR search. The PT number in GRRM output was used as a path timestamp. Compared to a typical random split, this splitting procedure allows to evaluate the ability of the model to generalize the information on the previously explored geometries toward geometries that will be explored in the future by the AFIR method.

Similarly, the training/validation set was split into a training set and a validation set by randomly splitting the earlier reactive events explored, with an 80%/20% ratio. The final training set and validation sets are then composed of the geometries corresponding

to these randomly splitted reactive events. This splitting procedure further focuses on the generalization from one reactive event to another.

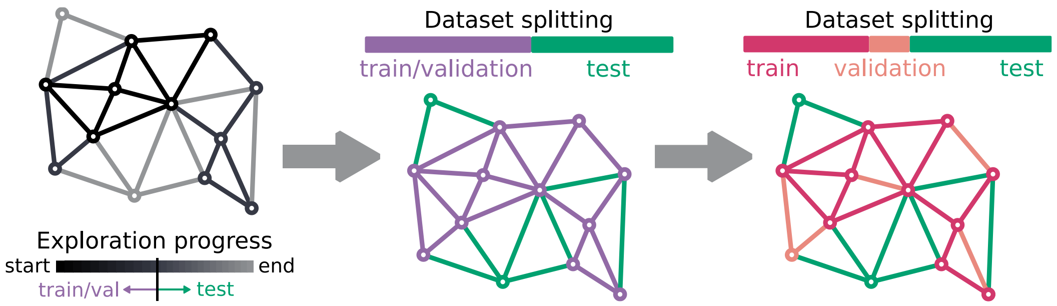

Figure S2. Dataset splitting scheme into training set, validation set and test set. First, a search-related timestamp is chosen (e.g., when 50% of the network's paths has been explored by the search, which is equivalent to: when the search is half-completed). The geometries corresponding to paths already explored before this timestamp are grouped in the train/validation set, and the test set is composed of all geometries corresponding to paths that were not yet discovered at this time of the search. The train/validation set is then splitted into a training set and a validation set randomly, while ensuring that all geometries corresponding to a single path are either within the training set or the validation set (i.e., validation geometries correspond to paths which are not covered in the training set, except for the EQs shared with training paths).

Three training/test sizes were considered:

- 16%/4%/80% training/validation/test split: In this setup, the training/validation is done on the first 1260 reactive events explored (~20%). At this stage, the most reactive reaction path retrieved so far differs significantly from the final converged path described in section 3.1: several important steps are missing.
- 40%/10%/50% training/validation/test split: In this setup, the training/validation is done on the first 3149 reactive events explored (50%). At this stage, the most reactive reaction path retrieved so far contains all the important steps identified in the final converged path, described in section 3.1. However, some energy barriers are not yet fully converged.
- 64%/16%/20% training/validation/test split: In this setup, the training/validation is done on the first 5038 reactive events explored (~80%). At this stage, the most reactive reaction path retrieved so far has already converged to the final path described in section 3.1.

S5. Influence of NNP training techniques on prediction performance

For each of the three training set sizes detailed section S4, several model features and training procedures were investigated to identify the most adapted ones to this particular application.

In a nutshell, among all the techniques tested, only Δ-learning provided significant improvements to the basic training scheme.

Influence of cold restarts

A cold restart is the action of re-training a model with the same data after it was already trained. By resetting the optimizer's parameters, this technique can help overcome training states being stuck in a local minimum.

Applying cold restarts did not significantly improved the Mean Absolute Error (MAE) on the predicted energies of the test set, independently of the training set size. Consequently, cold restarts were not applied in the rest of this study.

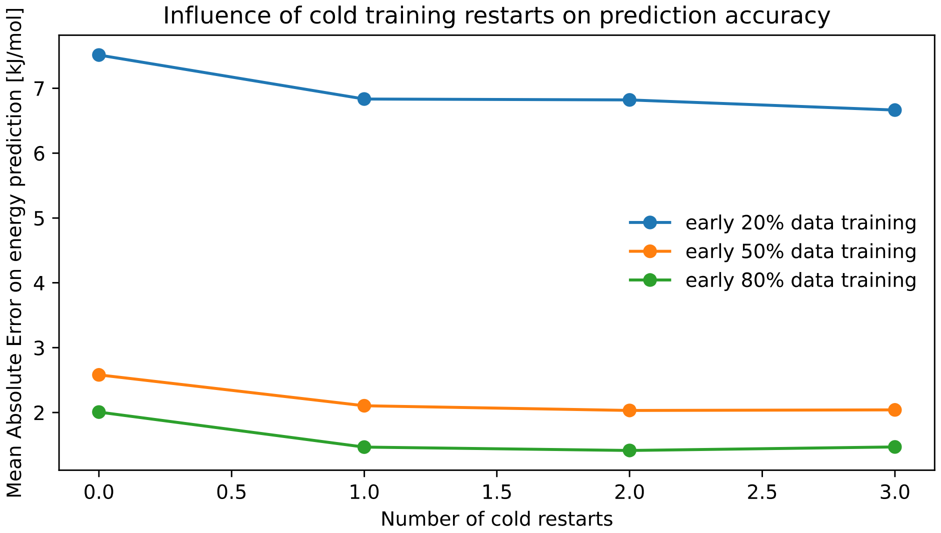

Figure S3. Influence of cold restarts on the MAE of predicted energies for the test set geometries, using different dataset splits.

Influence of dropout rate

SpookyNet's implementation support a specialized dropout mechanism to randomly reduce the number of interaction modules. This mechanism encourages the trained models to improve the efficiency of their convolutional filters, by retrieving non-local effects as much as possible with each interaction module application.

In practice, this mechanism did not provide consistent improvements on the test set performances of the trained models. Therefore, this mechanism was abandoned.

An alternative dropout strategy was also tested, in the spirit of the original dropout method [3], where each neuron has a user-defined probability of being dropped at each training step. In this alternative dropout approach, each interaction component has a user-defined probability of being dropped (the corresponding component of the interaction features vector is excluded from the cumulated features) during training. Inference is done with all components, weighted by the same probability value.

As previously, this alternative dropout strategy did not provide consistent improvements on the test set performances of the trained models, in practice. So, none of the dropout approaches tested were selected for the rest of this study.

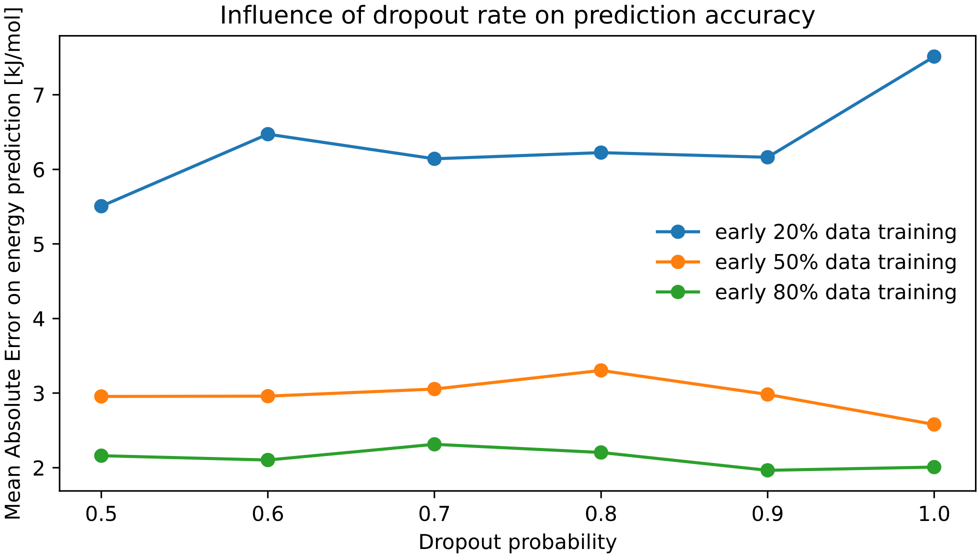

Figure S4. Influence of dropout rate on the MAE of predicted energies for the test set geometries, using different dataset splits

Influence of ensemble training

Ensemble training is a common solution to improve the accuracy and robustness of models at the cost of additional training time. In the ensemble training approach, the training/validation set is typically split into K mutually exclusive folds (i.e., sections). For each of these folds, a distinct model is validated on this fold and trained on the remaining K-1 folds. Therefore, K different models are generated, and the ensemble prediction is taken as the average of the K individual predictions.

Here, a 5-fold ensemble training was performed, and the ensemble prediction did not offer a significant improvement of the performances on the test set, compared to a single model.

Additionally, the disagreement between each K model predictions can be used as an estimator for the uncertainty of the ensemble prediction. We have then investigated the empirical correlation between the standard deviation of the ensemble models predictions and the actual prediction error of the mean ensemble predictions, for the energy. We found correlation coefficients of 0.52 for the 20%/80% train/test split, 0.66 for the 50%/50% train/test split, and 0.65 for the 80%/20% train/test split. Because linear correlation with prediction error is not particularly important for an uncertainty estimator, we have also studied the rank correlation with Kendall's τ coefficient implementation from the SciPy package. Kendall's τ coefficient is related to the probability that higher/lower ensemble disagreements correspond effectively to higher/lower prediction errors, respectively. The results are compiled in Table 2. Even though we found a clear statistical correlation, the ensemble disagreement cannot be considered in this case as a reliable estimator for the ensemble prediction uncertainty.

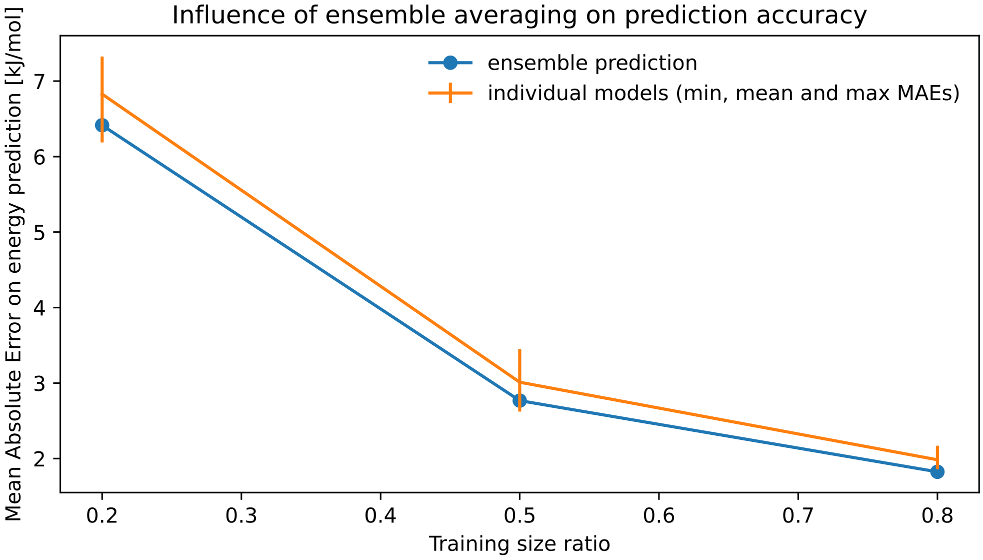

Figure S5. Comparison of 5-fold models ensemble and individual models on the MAE of predicted energies for the test set geometries, using different dataset splits. The error bars represent the range of accuracies for each of the individual models, and the orange line represents the average performance.

Table S1. Relation between ensemble disagreement (variance of ensemble prediction) and prediction error (error on mean prediction) on energies. Relations are studied on test set predictions, with 3 different train+validation/test splits. Concordance probability is the proportion of data pairs where a higher (resp. lower) ensemble disagreement matched a higher (resp. lower) prediction error, defined as $p = \frac{\tau+1}{2}$.

**Train+val/Test split**	**SpookyNet 20%/80% split**	**SpookyNet 50%/50% split**	**SpookyNet 80%/20% split**
Correlation coefficient	0.52	0.66	0.65
Kendall's τ coefficient	0.44	0.27	0.24
Concordance probability	0.72	0.64	0.62

Influence of additional terms

In addition to a Graph Convolutional Neural Network architecture, SpookyNet models also include additional terms by default. So, the predicted energy (gradients and Hessian predictions are analytically derived from the energy) is composed of 4 terms:

$$\mathrm{E}_{\mathrm{SpookyNet}} = \mathrm{E}_{\mathrm{NN}} + E_{ZBL} + E_{D4} + E_{elec}, \quad (1)$$

where E_{NN} is the Neural Network based atomic energy predictions, E_{ZBL} is a repulsion energy term from a Ziegler-Biersack-Littmark (ZBL) potential with learnable parameters, E_{D4} is the D4 dispersion correction term from Grimme et. al, and E_{elec} is an electrostatic term using partial charges predicted along with the atomic energies.

Such approach can be seen as an instance of Δ-learning, where model predictions are complemented by an external potential, so that the model can focus on learning only the difference (hence the Δ-learning name) between the target property and the external potential.

We have investigated the importance of these additional terms for obtaining accurate predictions, and we found that these additional terms did not provide improvements, per se, on the test set prediction accuracy.

Interestingly, we found that replacing these additional terms (ZBL repulsion, D4 dispersion and electrostatics) with predictions from a semiempirical potential (GFN2-xTB) led to significant improvements on the prediction capabilities, despite the xTB potential parameters being non trainable.

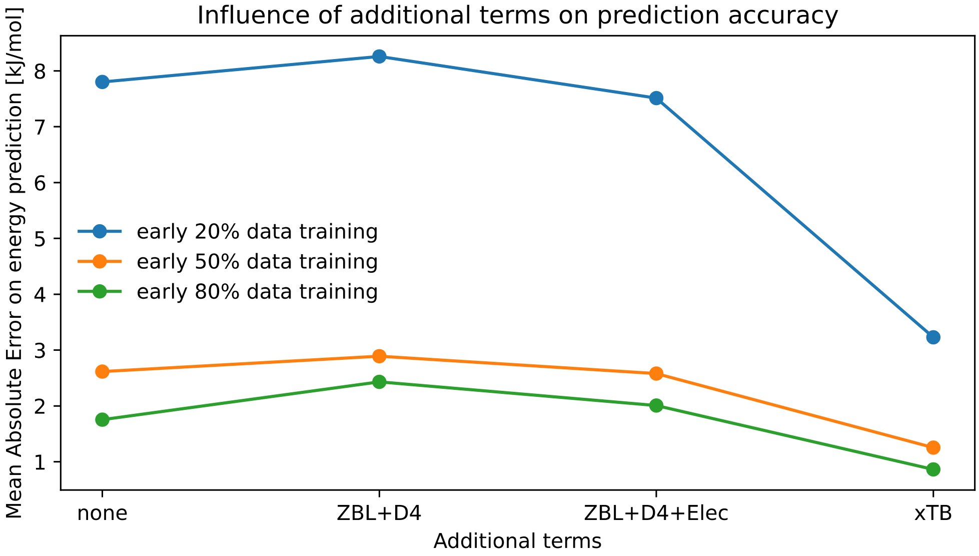

Figure S6. Influence of additional terms on the MAE of predicted energies for the test set geometries, using different dataset splits.

S6. Predicted main products

Table S2. Predicted main product (and corresponding yield) at different temperatures, from AFIR-based reaction path search using different potentials.

**Predicted main product + yield**	**GFN2-xTB**	**NNP(+xTB) 20% training**	**NNP(+xTB) 50% training**	**NNP(+xTB) 80% training**	**DFT**
250 K	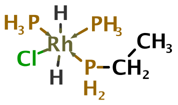 90.92%	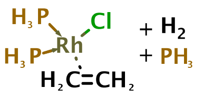 41.73%	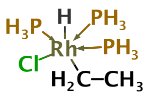 96.59%	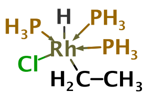 67.01%	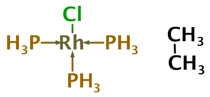 98.47%

300 K	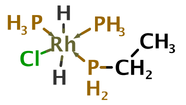 98.48%	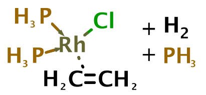 41.55%	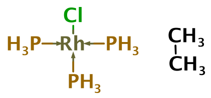 96.47%	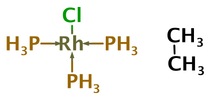 100%	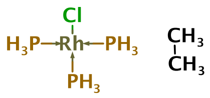 100%
350 K	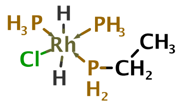 97.21%	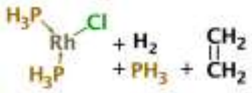 82.91%	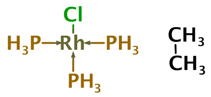 99.95%	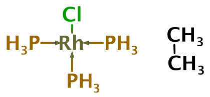 99.98%	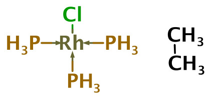 100%

Interestingly, even when NNP(+xTB) are mistaking the actual product, the predicted main product is still close to the species involved in the reaction. In constrast, GFN2-xTB consistently provides a chemically unreasonable main product (where the ethylene is bonded to a PH3 ligand).

Additionally, at low temperature (250K), the main product found by the sufficiently trained NNP(+xTB) models corresponds to the only side product found at DFT level (with the remaining 1.53% yield).

S7. Leaky holes behavior

Far from the training domain, we observe a "leaky holes" behavior around unphysical geometries, from insufficiently trained NNP(+xTB) models (using only the first 20% of the paths explored during the DFT-powered search). As it can be seen from Figure S7, these "leaky holes" are caused by the NNP part, unable to recognize broken geometries (very high energy regions). For example, the two most severely affected geometries contain steric clash within the H_2 molecule (H-H bond length ~ 0.37 Å). We believe this results from random artifacts, in the absence of neither training data nor physics-based guidance.

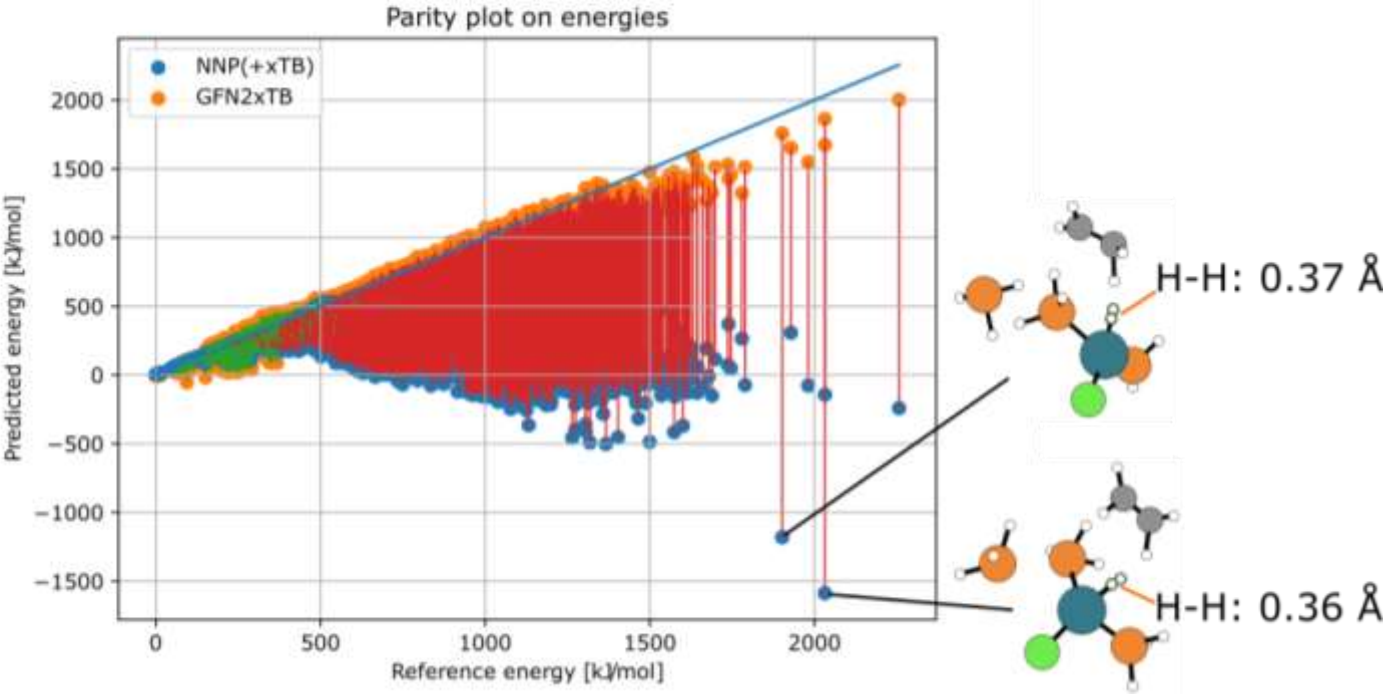

Figure S7. Performance of NNP(+xTB) models when powering a (global) AFIR-based exploration. Each point represents a PES stationary point geometry (i.e., an approximate TS or EQ) obtained during the search. The energy predictions are generated during the search, and the energy references (i.e., DFT energies) are computed a posteriori. xTB and NNP(+xTB) predictions for the same geometry are connected by a line: a red line if xTB only is closer to DFT, and a green line if the NNP contribution is beneficial. The energies potentials are shifted to match each others on the WilkinsonAFIRdb dataset. The model was trained on the first 20% of paths explored during the preliminary DFT-powered search.

This behavior begins to be visible even on the local AFIR-based search powered by the same NNP(+xTB) model (i.e., trained only on the first 20% of paths explored during the DFT-powered search), with a single high-energy geometry where the NNP part is severely mistaken, leading to an overall underestimated energy. This can be identified in Figure 12a, as the only large red line. The

corresponding geometry is represented Figure S8. Similarly to the most affected geometries from the global search, we observe a moderate steric clash, with a H_2 bond length of 0.6 Å. The fact that such broken geometries are explored even in the local search is illustrating the high-dimensionality of the chemical space, and its inherent challenges: even within the most sampled region of the reaction path network, unexplored broken geometries are accessible.

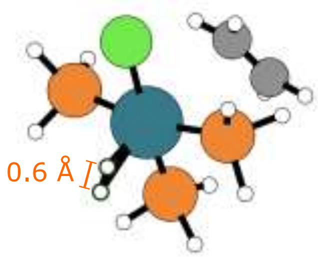

Figure S8. Unstable geometry wrongly identified as equilibrium state during the local AFIR-based search powered by a NNP(+xTB) model trained on only the frist 20% of paths explored during the preliminary DFT-powered AFIR-based search.

S8. Discussion

In this article, we are reporting a challenging, yet desirable, application of NNPs: to support kinetic studies involving transition metals complexes, via the AFIR method. The challenges of such application are illustrated by the surprisingly poor performance of state-of-the-art SpookyNet models for supporting an AFIR-based reaction path search, despite the same model displaying satisfactory performances on the geometries already obtained by a preliminary DFT-based AFIR search on the same system.

A difference in performance implies a difference in the geometries considered: we argue that the strong external forces applied during the search are naturally driving the system toward broken geometries, whereas the model is trained on chemically reasonable geometries. We therefore believe that the challenges encountered for NNP-powered AFIR-based searches arise from such fundamental separation between the model training domain and the application domain, due to the large external forces applied during optimization tasks as the search is performed.

However, the use of external forces is a core concept behind the AFIR method, allowing for efficient and automatic reaction path search, hence the need for models that can support those strong external forces. In addition, we cannot easily predict the application domain (i.e., toward which geometries the AFIR forces will drive the system into) a priori, and we argue that it would be unreasonable to try to sample the whole chemical space of broken geometries. Therefore, we are focusing here on expanding the applicability domain (i.e., where the model is performing properly) of NNPs from their training domain toward broken geometries, to hopefully englobe the application domain related to an AFIR-based search (see Figure 14). In other words, to design models that are able to extrapolate towards broken geometries despite not being trained on those.

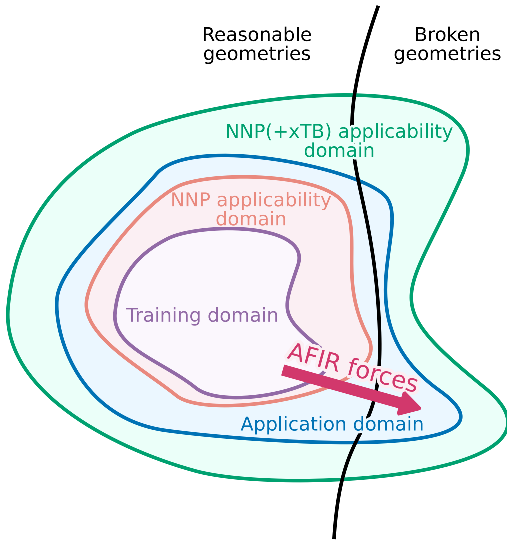

Figure S9. Illustration of the different domains of interest in the presence of AFIR forces. The training domain is the chemical space covered by the training data. The applicability domain is the chemical space where the model considered provide acceptable predictions. Here, the application domain correspond to the chemical space explored during an AFIR-based search.

Indeed, we observed that the tested SpookyNet models failed to extrapolate towards broken geometries. We believe that this lack of robustness is shared among the currently available general-purpose NNPs, because we blame it on the lack of physics in their functional form (i.e., mathematical model). Actually, recent efforts were made to add more physics-inpired terms [4].

In this regard, SpookyNet is a representative example of these efforts, as it includes physics principles though trainable additional terms with physics-inspired functional form. We believe the use of additional terms is in fact an appropriate way of including physics while keeping a desirable freedom on the neural network part. However, we found that the additional physics-inspired trainable terms of SpookyNet are simply not enough, as is, to achieve the level of robustness that AFIR searches require. Actually, in our quest for an appropriate solution, we concluded that there is no need for accurate extrapolation towards broken geometries because they are simply not chemically meaningful anyway. We argue that adding a simple safeguard to detect broken geometries is sufficient. A convenient way to do so is through Δ-learning, by adding (or, as it is the case with SpookyNet, by replacing the already existing physics-based terms) a single physics-inspired additional term, acting as a safeguard. This additional model needs to be both fast (to keep up with the neural network part) and robust (to identify broken geometries as such).

In that regard, we argue that a standalone external model is appropriate, which do not require training, acting as a universal potential. We found a semiempirical method such as GFN2-xTB to be well-adapted for such use.

Instead of designing yet another specialized NNP architecture, we emphasize that our proposed solution is model-agnostic and easily applicable, with a clear future-oriented design in a context of rapidly evolving NNP architectures.

For the choice of the external model, however, we believe that a simpler and faster model could be found, since accuracy is not required (i.e., a physically correct asymptotic behavior should be sufficient).

In any case, we found the NNP(+xTB) model to provide a local extension of the applicability domain around the training data, compared with a pure SpookyNet model. This improved extrapolation power towards broken geometries enables the first reported successful NNP-powered AFIR search, on the condition that sufficient training data is available. Indeed, we interpret that predicted reaction yields are acceptably reproduced (i.e., no "leaky holes" or underestimated energy barriers are unphysically capturing the reaction yield) when the training data is well sampled around the kinetically relevant reaction path network (that is expected to be explored during the AFIR search).

In that regard, we found GTM to be a promising visualization tool for reaction path networks, as the global NNP(+xTB) performances incidentally matched the GTM-based observations of training set completeness, in this particular case. Such observation raises the question of the applicability of GTM visualization to detect sufficient exploration during the construction of the preliminary training set.

Finally, while the amount of training data required for the specific example reported here is quite large (around 50% of a previous DFT-based study on the same system), we would like to emphasize that the main purpose of this study is merely to identify the challenges for NNP-powered kinetics studies and laying the foundations of robust NNP-based models compatible with the AFIR method.

Nonetheless, we believe one can easily reduce the amount of training data required for successful NNP(+xTB)-powered AFIR search, especially since the preliminary DFT-based study presented here was not specifically designed for efficiently building a training set.

For that purpose, we provide the reader with several ways of practical improvements:

- For example, in light of the results obtained in this study, a coarse AFIR-search (focusing on exploration, less refinement steps and larger sampling gaps) would have likely been sufficient.
- In addition, from the preliminary DFT-based study, we only considered the final optimized geometries along the explored reaction path network. Such choice is justified by the fact that currently available AFIR search datasets are unlikely to include additional data. Nonetheless, it is important to note that a practical AFIR search involve many refinement steps, representing many DFT evaluations (around 10-20 optimization steps are considered for each final geometry) that could easily be used as well for training.

- Last but not least, we believe it is possible to extract training data from multiple previous AFIR searches via transfer learning, allowing to combine data from different systems. The idea behind this approach is to replace training data on the considered system by training data from multiple similar systems already available.

Outliers

Strong outliers were found with training on the first 20% of paths explored, however the outliers severity is drastically reduced using the first 50% or more of paths explored. This analysis is consistent with the GTM-based observation that the first 20% of paths does not cover the full reaction path network.

However, we believe that these outliers are not responsible for the poor performance of the SpookyNet models during the AFIR-based search. Instead, we attribute this performance to strong energy underestimations and a "leaky hole"-like behavior due to the inherent poor description of broken geometries.

AFIR-based search acceleration scheme

For this study, we have considered the same system for both the training and inference of the NNP model. In other words, the model is used for predictions on conformers of the training geometries. Such scheme is adapted to reduce the cost of a complete AFIR search, by performing only a short preliminary exploration of the reaction path network, then performing the actual full search using an NNP model trained on the preliminary exploration data. This simple approach generates specifically trained models, and accelerates in principle the full kinetic study by exploring, with expansive ab initio calculations, only a fraction of the reaction path network.

In addition, such scheme could also be used to run a retrosynthetic AFIR-based search from a forward search, allowing to explore alternative substrates that are promising to obtain the targeted product.

π-acidity of the PH_3 ligands

It is known that the simulated π-acidity of the PH_3 ligands is particularly sensitive to the accuracy of the P 3d orbitals description, resulting in drastic π-acidity decrease in the absence of P 3d orbitals [5]. We have therefore investigated briefly the effects introduced by considering a Def2-TZVP basis set instead of Def2-SVP. We observed only moderate effects, translated into changes in the energy barriers up to 20 kJ/mol, without much impact on the reaction path, so our finding do not seem to be qualitatively affected. Anyway, such analysis is not the main topic of this article.

S9. Detailed conclusion and perspectives

Ab initio kinetic studies are important to understand and design chemical reactions. While the kinetic-based navigation method provides an efficient and automatic framework to search reaction paths, such studies still typically require a large amount of ab initio calculations, due to the combinatorial nature of the reaction path network exploration. As an illustration, the AFIR-based search reported section 3.1 required more than 1.8M gradients calculations in total. In this context, performing the full search at a high accuracy level of theory can incur large computational costs.

In this article, we have investigated the possibility to replace expensive ab initio calculation with fast NNP predictions, during the search, to accelerate such kinetic studies. For this purpose, we have considered the hydrogenation of ethylene catalyzed by a transition metal complex inspired by Wilkinson's catalyst. In this example, where the transition metal plays a major role, we found that GFN2-xTB is not accurate enough to reproduce the reaction kinetics, therefore misleading the kinetic-based navigation.

For this investigation, we have first performed a preliminary reaction path search at a DFT level of theory, using the AFIR method with kinetic-based navigation. This novel theoretical study, reported in section 3.1, extends the prior study from Koga *et al.* [6] by explicitly considering all three PH3 ligands of the catalyst.

The resulting reaction path network's data was visualized using GTM, with sorted-pairwise distance descriptors and Boltzmann-like weighting to encode an ensemble of 3D structures generated during the search. The energy and class GTM landscapes allowed to follow the exploration of the chemical space during the search and to identify the zones corresponding to the different steps of the reaction.

The geometries obtained in the early stages of the aforementioned DFT-powered reaction path search were used to train NNP-based models intended for supporting a reaction path search on the same system. Such NNP-powered AFIR-based search was made possible for the first time, using our newly developed GRRM-NNP interface.

Despite excellent prediction accuracy on pre-obtained geometries (i.e., on data obtained from the DFT-based search), SpookyNet models displayed very poor performances when supporting an AFIR-based search, regardless of the training set size.

The specificity of the AFIR method lies in the presence of large external forces, prone to the generation of broken geometries if the supporting model is not robust. This result illustrates the failure of general-purpose NNPs to handle broken geometries when trained on reasonable geometries only.

We solved this robustness issue by complementing NNP predictions with a robust semiempirical model such as xTB, acting as a safeguard against leaky holes and unrecognizing broken geometries.

The resulting NNP(+xTB) models were found capable of supporting an NNP-powered AFIR-based search, successfully reproducing the reaction yield, as long as their training set is representative of the reaction path network explored during the search.

In that regard, GTM might be used to check the completeness of training sets, but further studies are required to establish such an application.

Furthermore, while the present article focused on reporting the challenges of using NNPs for supporting AFIR-based reaction path search, we believe that it is possible in practice to dramatically reduce the amount of training data required for a dedicated application (see the guidance provided in section S8). In particular, transfer learning would be a fitting solution to make use of available data on similar systems.

Much work was accomplished over the years to design efficient and automatic reaction path search procedures, such as the AFIR method with kinetic-based navigation. Still, efficient exploration of the PES fundamentally relies on PES evaluations that are fast, accurate and robust, even in the presence of transition metal catalysts. As such, we believe that kinetic studies can benefit much from the recent developments in the NNP field, and the promising performances of our NNP(+xTB) solution is highlighting the importance of robustness for designing adapted potentials.

S10. Additional GTM-related analyses

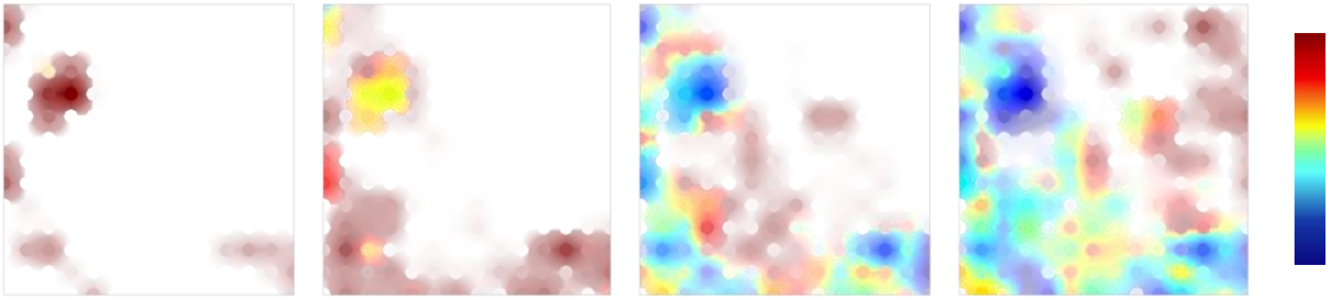

Figure S10. GTM one-class landscapes where the first 0 to 1%, 1 to 5%, 5 to 10% and 10 to 20% of the network explored during the DFT-based search are projected and compared. Blue area indicates map area containing structures already projected at the lowest percentage, while red area are area where recently discovered structures are projected.

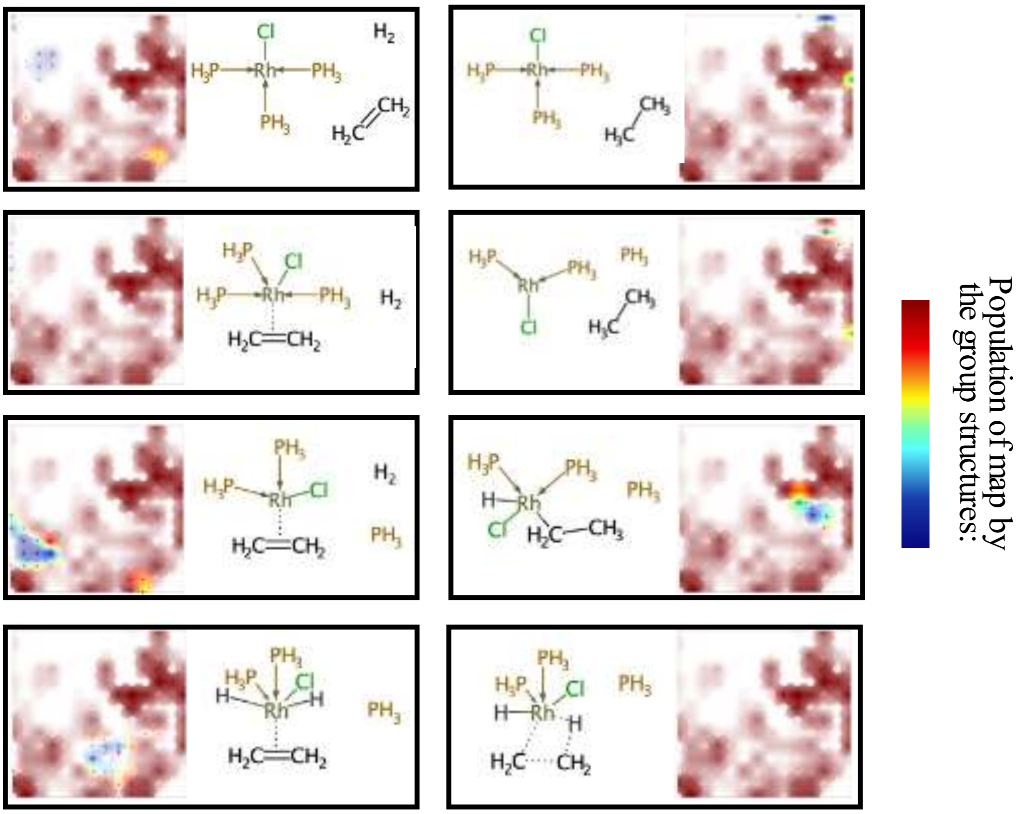

Figure S11. Class landscapes describing distribution of 3D structures and corresponding 2D structures for main reaction steps 1-6 (see also Figure 6).

S11. Methods – used software

Marvin was used to draw and display chemical 2D structures [7] [Marvin version 23.2, ChemAxon (https://www.chemaxon.com)]. 3Dmol was used to display chemical 3D structures [8]. The ASE python package [9] was used for 3D visualization and database management. Plots were generated using matplotlib [10].

References

1. Sumiya, Y.; Maeda, S. Rate Constant Matrix Contraction Method for Systematic Analysis of Reaction Path Networks. *Chem. Lett.* **2020**, *49*, 553–564, doi:10.1246/cl.200092.
2. Unke, O.T.; Chmiela, S.; Gastegger, M.; Schütt, K.T.; Sauceda, H.E.; Müller, K.-R. SpookyNet: Learning Force Fields with Electronic Degrees of Freedom and Nonlocal Effects. *Nat. Commun.* **2021**, *12*, 7273, doi:10.1038/s41467-021-27504-0.
3. Srivastava, N.; Hinton, G.; Krizhevsky, A.; Sutskever, I.; Salakhutdinov, R. Dropout: A Simple Way to Prevent Neural Networks from Overfitting. *J. Mach. Learn. Res.* **2014**, *15*, 1929–1958.
4. Käser, S.; Vazquez-Salazar, L.I.; Meuwly, M.; Töpfer, K. Neural Network Potentials for Chemistry: Concepts, Applications and Prospects. *Digit. Discov.* **2023**, *2*, 28–58, doi:10.1039/D2DD00102K.
5. Pacchioni, G.; Bagus, P.S. Metal-Phosphine Bonding Revisited. .Sigma.-Basicity, .Pi.-Acidity, and the Role of Phosphorus d Orbitals in Zerovalent Metal-Phospine Complexes. *Inorg. Chem.* **1992**, *31*, 4391–4398, doi:10.1021/ic00047a029.

6. Koga, N.; Daniel, C.; Han, J.; Fu, X.Y.; Morokuma, K. Potential Energy Profile of a Full Catalytic Cycle of Olefin Hydrogenation by the Wilkinson Catalyst. *J. Am. Chem. Soc.* **1987**, *109*, 3455–3456, doi:10.1021/ja00245a044.
7. Marvin version 23.2, ChemAxon (https://www.chemaxon.com)
8. Rego, N.; Koes, D. 3Dmol.Js: Molecular Visualization with WebGL. *Bioinformatics* **2015**, *31*, 1322–1324, doi:10.1093/bioinformatics/btu829.
9. Hjorth Larsen, A.; Jørgen Mortensen, J.; Blomqvist, J.; Castelli, I.E.; Christensen, R.; Dułak, M.; Friis, J.; Groves, M.N.; Hammer, B.; Hargus, C.; et al. The Atomic Simulation Environment—a Python Library for Working with Atoms. *J. Phys. Condens. Matter* **2017**, *29*, 273002, doi:10.1088/1361-648X/aa680e.
10. Hunter, J.D. Matplotlib: A 2D Graphics Environment. *Comput. Sci. Eng.* **2007**, *9*, 90–95, doi:10.1109/MCSE.2007.55.
